# Gastric Emphysema Induced by Severe Vomiting

**DOI:** 10.7759/cureus.6487

**Published:** 2019-12-28

**Authors:** Amal Ghneim, Sreenath Meegada

**Affiliations:** 1 Internal Medicine, The University of Texas Health Science Center/Christus Good Shepherd Medical Center, Longview, USA

**Keywords:** intractable vomiting, air, stomach, emphysema

## Abstract

Gastric emphysema or air in the stomach wall is a rare condition resulting from the disruption of gastric mucosa and entry of air into the stomach wall which could be from severe vomiting, instrumentation or endoscopy, gastric ischemia, and dissection of air from the mediastinum. Treatment is usually conservative and supportive. We report the case of a 70-year-old woman with a three-day history of nausea and vomiting who presented with gastric emphysema on computed tomography (CT) imaging; she responded very well with conservative treatment and was discharged in a stable condition.

## Introduction

Gastric pneumatosis is defined as the presence of air in the stomach wall. It is a rare radiological finding, and there are fewer than 50 cases described in the literature so far [1]. It is classified as i) gastric emphysema, which is defined as gas in the gastric wall that comes from the environment and ii) emphysematous gastritis, which is defined as gas that is produced within the wall [2]. These present differently and carry vastly different mortality rates and outcomes.

## Case presentation

**Figure 1:**
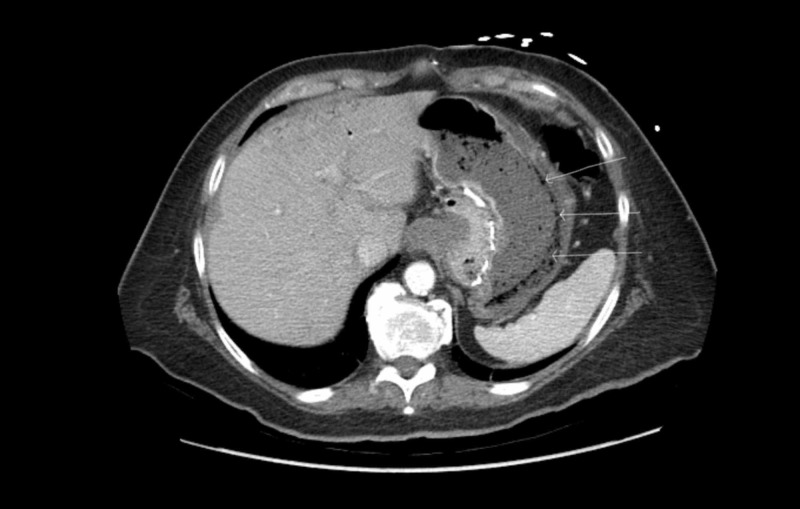
Computed tomography (CT) of the abdomen showing gastric emphysema (arrows)

**Figure 2:**
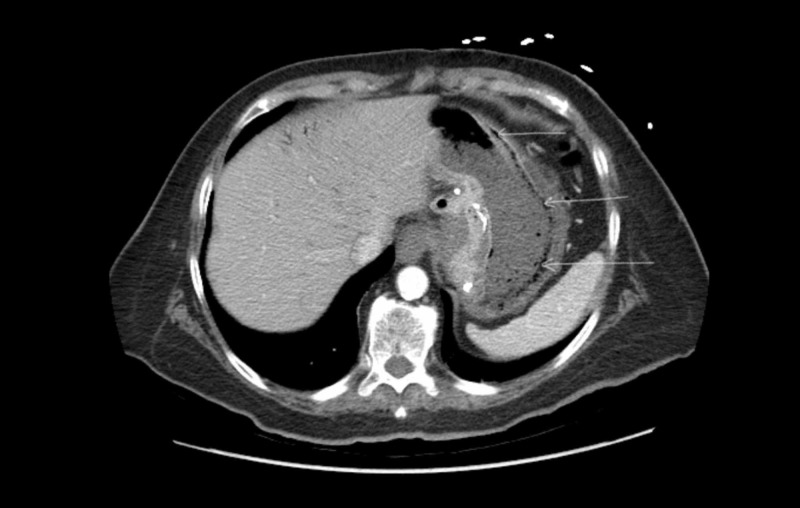
Computed tomography (CT) of the abdomen (different slice) showing gastric emphysema (arrows)

A 70-year-old Caucasian female presented to our facility from a nursing home with complaints of nausea, vomiting, and diarrhea for three days. She has a past medical history of vertical band gastroplasty, hypertension, bipolar disorder and traumatic brain injury that resulted from a gunshot wound to her head over 20 years ago. She initially presented for concerns of dehydration - her initial blood pressure was 111/56. In the emergency room, initial laboratory workup revealed an elevated white blood cell count of 14,700 cells per cubic millimeter of blood with 72.5% neutrophil differential, normal electrolytes, and a creatinine of 1.07 which was increased from her baseline. She appeared clinically dehydrated and was complaining of nausea and abdominal pain. She was started on intravenous (iv) normal saline for rehydration. Computed tomography (CT) of the abdomen and pelvis with IV contrast was ordered. It showed wall thickening of the stomach with gas within the gastric wall (Figures 1-2).

The imaging was not able to discern between gastric emphysema and emphysematous gastritis. Small bowel and colon were visualized and were normal. Findings were immediately discussed with the general surgeon and given the patient’s poor functional status as well as mild symptoms, she was deemed not a surgical candidate. She was continued on IV fluid resuscitation as well as started empirically on IV meropenem. She was followed closely that night with serial abdominal exams. She remained stable with improvement in blood pressure and creatinine. She was observed in our hospital for three days. Her diet was advanced slowly, and she was able to tolerate a regular diet with no nausea or vomiting. Her abdominal exam remained benign. Given the resolution of her symptoms, she was discharged back to her nursing home with a complete resolution of her symptoms.

## Discussion

Gastric emphysema carries an excellent prognosis with conservative management [1]. Most patients present with mild or no symptoms as our patient. Gastric emphysema results from the disruption of gastric mucosal wall that leads to air entry into the wall. Both extrinsic and intrinsic factors can cause this. Extrinsic examples include trauma via instrumentation with upper gastrointestinal endoscopy or open abdominal surgery. Intrinsic causes include increased intra-gastric pressure from gastric outlet obstruction and severe vomiting or dissection of air from the mediastinum as with a pneumothorax. Ischemia can also cause gastric emphysema with the main difference being patients present with severe abdominal pain and an acute abdomen [1].

Emphysematous gastritis is more severe than gastric emphysema and carries a mortality rate of 61% [3]. Patients present with an acute abdomen and clear features of systemic toxicity and sepsis. Etiologies can include alcohol abuse, abdominal surgery, diabetes, or ingestions of corrosive substances [3]. It is different from gastric emphysema in that the gas is formed in the bowel and associated with bacterial infections. One study revealed that some of the involved organisms were Streptococci, Escherichia coli, Enterobacter species, Clostridium welchii, and Staphylococcus aureus [3]. Treatment is mostly supportive with parenteral antibiotics, IV fluid resuscitation, and vasopressors as needed [4]. Surgery is avoided during the acute phase because of the tissue friability, with gastrectomy being reserved only when medical management fails [5].

## Conclusions

Gastric pneumatosis is an uncommon radiological finding which can represent either gastric emphysema or emphysematous gastritis. These two conditions differ a lot in management and prognosis, so diagnosis should be made with a thorough history, physical examination, and clinical presentation. Our patient presented with a three-day history of severe vomiting; CT abdomen was positive for intraluminal gastric air and there were no signs of systemic toxicity. She did respond well with conservative treatment which led to the diagnosis of gastric emphysema.
